# The first finding of parasitic mite, *Parasteatonyssus nyctinomi* (Mesostigmata: Gamasina: Macronyssidae), in Namibia

**DOI:** 10.4102/jsava.v91i0.2002

**Published:** 2020-05-21

**Authors:** Maria V. Orlova, Theresa M. Laverty, Will K. Reeves, Elena M. Gratton, Mallory L. Davies

**Affiliations:** 1 Institute of X-Bio, University of Tyumen, Tyumen, Russia; 2 Laboratory of biodiversity monitoring, National Research Tomsk State University, Tomsk, Russia; 3 Department of Fish, Wildlife, and Conservation Biology, Colorado State University, Fort Collins, United States; 4 C. P. Gillette Museum of Arthropod Diversity, Museum Affiliate, Colorado State University, Fort Collins, United States

**Keywords:** *Parasteatonyssus nyctinomi*, Macronyssidae, *Tadarida aegyptiaca*. Namibia, Egyptian free-tailed bat

## Abstract

Sixty-four individuals of a macronyssid mite, *Parasteatonyssus nyctinomi* (Zumpt, Patterson 1951), were identified from Egyptian free-tailed bats *Tadarida aegyptiaca* (É. Geoffroy 1818) (Chiroptera: Molossidae) captured in the Kunene region of Namibia (southern Africa). This is the first report on *P. nyctinomi* in the country.

## Introduction

Species in the family Macronyssidae Oudemans 1936 include parasites of mammals, birds and reptiles. The more derived macronyssidae, subfamily Ornithonyssinae Lange 1958, are a relatively uniform group characterised by some specific morphological peculiarities (Radovsky 2010). Genus Parasteatonyssus was derived from the genus Ornithonyssus Sambon 1928 by Radovsky (1966) and includes bat ectoparasites (mostly parasitising free-tailed bats [Chiroptera: Molossidae]). The genus contains the following five species: *Parasteatonyssus cornutus* (Keegan, 1956), *Parasteatonyssus hoogstraali* (Keegan, 1956), *Parasteatonyssus jayanti* Advani, Vasirani, 1981, *Parasteatonyssus lingeraji* (Hiregaudar, Bal, 1956) and *Parasteatonyssus nyctinomi* (Zumpt, Patterson, 1951) (Radovsky 2010), but a lack of complete and comprehensive knowledge of the ecology of principal hosts of this genus – free-tailed bats (including their host–parasite relationship) – does not allow accurate conclusions about the systematics and distributions of these mites. Only two species (*P. cornutus* and *P. nyctinomi*) have been recorded previously in Africa.

This article presents new findings about *P. nyctinomi* from the Egyptian free-tailed bat, *Tadarida aegyptiaca* (É. Geoffroy, 1818) (Chiroptera: Molossidae), in Namibia.

## Materials and methods

The research was conducted on Namib Desert bats and their ectoparasites in the Kunene region of northwestern Namibia. Bats were captured from 06 December 2016 to 04 April 2017 by deploying mist nets, and captured individuals were examined intensively for ectoparasites. Bats were morphologically identified using taxonomic descriptions given by Monadjem et al. (2010). All ectoparasites were removed with forceps, pooled into one sample for each individual bat and preserved in 95% ethanol before the bats were released. For each ectoparasite sample, the mites were transferred into a new vial containing 70% ethanol; all mites were sent to the Institute of Environmental and Agricultural Biology (X-Bio) of University of Tyumen (Russia) for species identification. Mites were mounted on permanent slides with Faure–Berlese mounting medium (Whitaker 1988). Morphological identification of mites was performed by one of the authors (M.O.), according to the keys given by Micherdzinski (1980) and Radovsky (2010). Photographs were taken with a digital camera AxioCam ICc5 (Zeiss, Germany) via a compound microscope AxioImager A2 (Zeiss, Germany) with a phase-contrast and differential interference contrast (DIC) objectives. All measurements are given in micrometers (*μ*m).

Voucher specimens mounted on slides have been deposited at the collection centre of the University of Tyumen’s Museum of Zoology, Tyumen, Russia.

### Ethical considerations

All applicable institutional, national and international guidelines for the care and use of animals were followed. Fieldwork was conducted with in accordance with the guidelines of Colorado State University’s Institutional Animal Care and Use Committee (Protocol #15-6140A) and the Ministry of Environment and Tourism, Republic of Namibia (research/collecting permits #2122/2016 and #2225/16).

## Results

Sixty-four specimens of macronyssid mites were obtained from four Egyptian free-tailed bat, *T. aegyptiaca*, individuals in three localities: Spaarwater Pos (23° 68’ S, 15° 19’ E) (20 individuals), Sesfontein (19° 12’ S, 13° 62’ E) (14 individuals) and Hoanib Skeleton Coast Camp (19° 36’ S, 13° 15’ E) (30 individuals).

**FIGURE 1:**
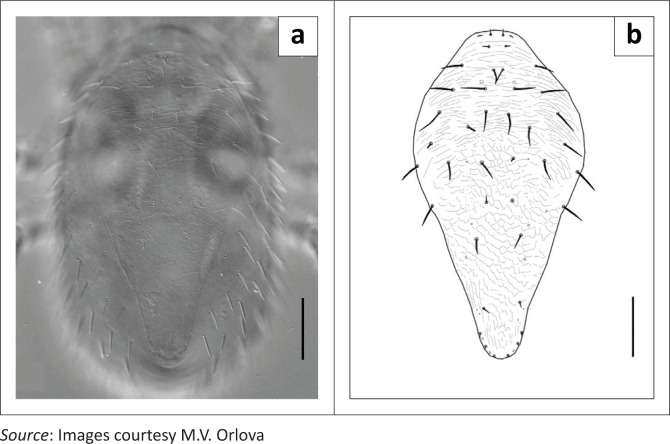
*Parasteatonyssus nyctinomi*, ♀: (a) photograph, dorsal view; (b) drawing, dorsal shield. Scale bar: 100 *μ*m.

**FIGURE 2:**
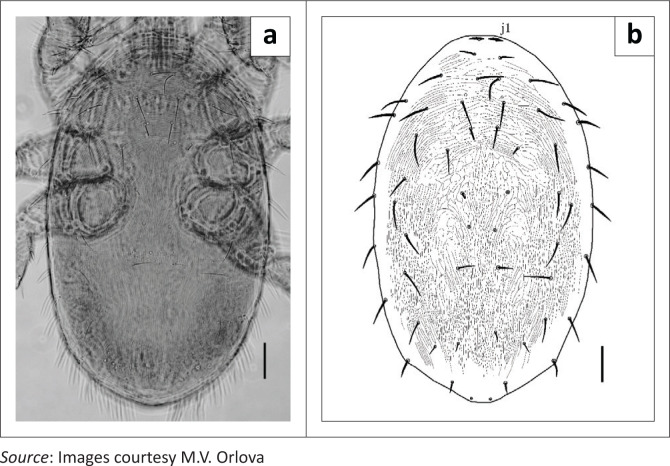
*Parasteatonyssus nyctinomi*, ♂: (a) photograph, dorsal view; (b) drawing, dorsal shield. Scale bar: 50 *μ*m.

Mites were morphologically identified as 41 females (Figure 1), seven males (Figure 2) and 16 protonymphs of *P. nyctinomi* (Zumpt and Patterson, 1951).

## Discussion

This is the first report from Namibia on *P. nyctinomi*. To our knowledge, previous findings providing the first description of *P. nyctinomi* are known from South Africa (Zumpt, Patterson, 1951). *Type host* of *P. nyctinomi was Nyctinomus bocagei* Seabra 1900 (Chiroptera: Molossidae) (Zumpt, Patterson, 1951) – a junior synonym (a name which describes the same taxon as a previously published name) of *T. aegyptiaca*. The Egyptian free-tailed bat is widely distributed throughout Africa, except parts of the northwest and east through Arabia and the Middle East to southern Asia as far east as Bangladesh and south to Sri Lanka (Skinner, Chimimba, 2005). Accepting that *P. nyctinomi* is a specific ectoparasite of *T. aegyptiaca* is true, we can expect new records of the mite in the Palearctic part of Africa, Arabia and India where *T. aegyptiaca* is endemic. The lack of records is most likely because of collection difficulties. Because of a lack of knowledge about the life cycle of *P. nyctinomi*, more studies are necessary to determine the principal host and geographic distribution of this parasite species.
